# Clinical Evaluation of Herbal Medicine (ICH-012) in Treating Acute Cerebral Haemorrhage: Safety and Efficacy from 6- to 72-Hour Time Window (CRRICHTrial-II)

**DOI:** 10.1155/2018/3120179

**Published:** 2018-08-26

**Authors:** Qixin Zhang, Liling Zeng, Xiuyan Chen, Yuexiang Zhou, Baoying Gong, Haijun Li, Jianwen Guo

**Affiliations:** Brain Center, The Guangdong Provincial Hospital of Chinese Medicine, The Second Teaching Hospital of Guangzhou University of Chinese Medicine, China

## Abstract

**Background:**

Hypertensive intracerebral haemorrhage (HICH), which is characterized by rapid change, high morbidity, and mortality, is extremely dangerous. Both medical and surgical treatments lack definitive evidence and remain controversial. A prospective RCT that we have conducted has shown that the usage of the herbal medicine ICH-012 within 6 h of the event may increase the risk of haematoma enlargement and gastrointestinal bleeding. However, the volume of haematoma remains stable after 6 h. Thus, we will increase the time window to the period from 6 to 72 h after onset to evaluate the safety and efficacy of ICH-012 treating ICH (ClinicalTrial.gov ID: NCT03354026).

**Methods/Design:**

The CRRICHTrial-II study, a prospective, double-blinded, controlled, multicentre RCT, includes three groups: A, B, and C. Group A patients were treated with 8 herbal medicines (with 2 herbal medicines of Hirudo and Tabanus as well as 6 other combined herbal medicines of Group B) and Group C were placebo. Patients should meet all the inclusion criteria: age between 18 and 80 and diagnosis of HICH by brain CT scan between 6 and 72 h from the onset. The CT scan will be taken at four critical time points: baseline, between 6 and 72h, 24h after onset, and between 10 and 14 days after onset. The drug intervention lasts 10 days, and there is a follow-up visit taken after 90 days. The haematoma enlargement after 24 h onset as demonstrated by CT is the primary outcome.

**Discussion:**

A large amount of data from high-quality RCTs is needed for the extensive clinical application of herbal medicine. The CRRICHTrial-II will evaluate the safety and effectiveness of ICH-012 in a safer time window between 6 and 72 h and investigate the possible mechanisms of action and direction of herbal medicine in the haematoma growth after HICH.** Trial registration at **ClinicalTrial.gov, ID: NCT03354026, is registered on 23rd Nov. 2017.

## 1. Update

### 1.1. Background

Cerebrovascular disease has become the first leading cause of death in Chinese people, and haemorrhagic stroke (intracerebral haemorrhage, ICH) is one of the most important manifestations [1]. Prevention and standardized treatment are critical issues worldwide because of the rapid onset, rapid progress, high morbidity, and rates of disability for ICH [2, 3]. It is generally believed that the enlargement of haematoma after ICH is an important factor of poor prognosis [4, 5]. Moreover, as an independent risk factor for stroke prognosis, the enlargement of haematoma is shown to be closely related to some clinical indicators including the presence and number of CT spot sign number, hypertension, and coagulopathy [6–8]. In recent years, the genotype of ApoE 2 has been discovered to be related to the rupture of vessel walls in amyloid cerebrovascular disease as well as the haematoma enlargement of lobar haemorrhage; however, genetic testing to predict haematoma enlargement is still in the exploratory stage [9–11]. Unfortunately, neither medical nor surgical clinical trials have had positive results for the HICH treatment. Thus, we have no medications or surgical methods to reduce the mortality and morbidity of HICH patients [12–14].

In China, the curative effects of the traditional Chinese medicines for ICH have been demonstrated clinically, which plays an important role in reducing the mortality and disability of ICH. Herbal medicine that promotes blood circulation and reducing blood stasis was most commonly used in clinical practice [15–17]. On the other hand, according to research we have conducted a prospective, randomized, double-blind, controlled clinical trial (Trial registration clinicaltrials.gov: NCT01918722) in which the herbal medicine ICH-012 may increase the risk of haematoma growth and gastrointestinal bleeding without efficacy in reducing mortality or morbidity during the 6 h time window [18]. We may delay the time window of the drug delivery to 6-72 h onset at the next stage in consideration of the high incidence rate of haematoma enlargement within 6 h. Furthermore, we must increase the sample size to more than 360 individuals.

## 2. Methods/Design

### 2.1. Objectives

This trial's primary aim is to evaluate the safety and efficacy of the herbal medicine ICH-012 in treating AICH within the time window of 6 to 72 h from onset. Exploring the possible mechanisms of action of the herbal medicine is also our concern.

### 2.2. Study Design

**Figure 1:**
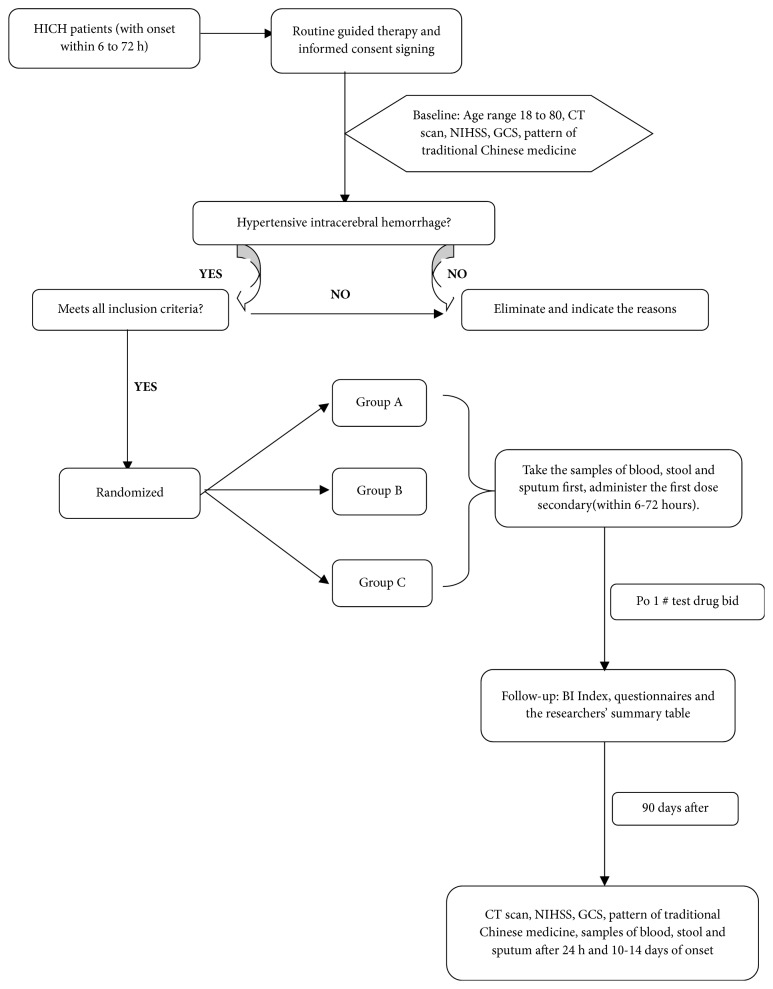
The flowchart of the CRRICHTrial-II.

This is a prospective, randomized, double-blinded, controlled trial (ClinicalTrial.gov, ID: NCT03354026) involving 7 participating neurological centres. The flowchart of this research is shown in Figure 1. Patients with acute intracerebral haemorrhage diagnosed by brain CT scan within 6 to 72 h onset are included in the scope of screening first. The informed consent will be signed when the patient meets all the inclusion criteria shown in the following category of Eligibility Criteria. The trial includes three random allocation groups: Groups A, B, and C. All the enrolled patients will take the first dose of study medicine within 6 to 72 h of ictus before scoring NIHSS and GCS. The medicine intervention will last 10 days, while the CT scan will be taken at three critical time points: between 6 and 72 h, 24 h after, and between 10 and 14 days after. There is a follow-up visit taken after 90 days, during which the researchers will finish the questionnaires and summary table. The primary outcome is haematoma enlargement after 24 h onset demonstrated by CT, while the secondary outcomes include mortality and severe adverse events.

This trial will be conducted in the departments of neurosurgery at the following 7 Chinese hospitals: (1) The Second Teaching Hospital of Guangzhou University of Chinese Medicine, Guangzhou; (2) Lianjiang People's Hospital, Lianjiang; (3) Shenyang Second Hospital of Traditional Chinese Medicine, Shenyang; (4) The Third People's Hospital of Hubei Province, Hubei; (5) Liaocheng People's Hospital, Liaocheng; (6) Zengcheng Hospital of Traditional Chinese Medicine, Zengcheng; and (7) Cong Hua Hospital of Chinese Medicine, Conghua. Researchers from different hospitals will receive unified training and assessment before the beginning of the trial. Quality control of the trial will be undertaken by the subject team at least once a year.

## 3. Eligibility Criteria 

### 3.1. Inclusion Criteria

1. Men and women aged from 18 to 80 years
2. Acute cerebral haemorrhage confirmed by brain CT scan within 6 to 72 h from onset
3. Glasgow Coma Scale ≥6
4. Signed informed consent

### 3.2. Exclusion Criteria

1. Secondary cerebral haemorrhage caused by brain tumour, blood diseases, cerebrovascular malformation (anomaly), aneurysm, or other pathology
2. Patients with severe heart, liver, and renal insufficiency
3. Intolerance to traditional Chinese medicine (TCM) or history of allergies
4. Patients with severe cerebral hernia in the early onset stage
5. Poor compliance

### 3.3. Centre Eligibility

A total of 7 hospitals across China participated in this study, including the principal responsible unit. All the participation centres are equipped with qualified neurology centres, equipment, and specialists for standardized medical care and clinical trials.

## 4. Sample Size and Randomization

### 4.1. Sample Size

The enlargement percentage of the haematoma volume measured on the CT scan at the time 24 h after onset is the primary outcome of this study. We designed 3 groups (A, B, and C) to balance and calculate the enlargement percentage of the haematoma by blinded methods. The hypothesis is that haematoma in the three groups is not different. According to a previous literature review, the rate of haematoma enlargement at 24 h after onset is likely between 14% to 20% [19–21]. The Chinese herbal medicines used in treating AICH in the 3 groups associated with the trial are shown in Table 1 [17, 18]. With the parameters set at *α*=0.05, 1-*β*=80%, and the primary outcome rate=20%, we used the following formula to calculate the sample size. The loss rate is supposed to be approximately 10%, and 360 patients are necessary.

(1) n=1641.6γsin⋀−1√π_max−sin∧−1√π_min∧2γ  is  12.65,π_max  38%  and  π_min7%

### 4.2. Randomization

The Chinese medical herbs used here are from the Pharmacopoeia of the People's Republic of China released on 5 June 2015. Our randomized programme was completed by the key clinical research laboratory of the Traditional Chinese Medicine Hospital of Guangdong Province. We assigned 306 cases into three groups: Groups A, B, and C in the proportion of 1:1:1. Group A, the first experimental group, used RBS, which includes 8 herbal medicines, Hirudo and Tabanus, and 6 herbals of promoting blood circulation. Group B, the second experimental group, used an herbal medicine, which includes all the herbals in Group A except Hirudo, Tabanus, and rhubarb. Group C is a placebo group with dextrin, farina, and so on. This study adopts a stratified random sampling method and intra-slice randomization on the basis of using PROC PLAN progress in SAS V9.2. In addition, the experimental measures and the control measures will be double blind. The surface of the opaque randomization envelopes will indicate the information of the test name, hospital name, and the entry sequence number of the patient. The research process of incorporating the patient, dispensing medicine depending on the random envelopes, and others will be supervised by the researchers.

## 5. Intervention Treatment

The first dose will be arranged within 6 to 72 h of onset, and the medicine will be taken twice a day for 10 days. The details of the treatment intervention in the three groups are shown in Table 2. All our pharmaceutical preparations are produced by the Kang Yuan pharmaceutical company, which has qualified pharmaceutical production facilities in China. Usage of the medicine is as follows: dissolve each bag of medicine in 80 to 100 ml warm water and take orally or receive through nasal feeding by the researchers. Each time the medicine is administered, the researchers must record it and implement quality control.

### 5.1. CT Scanning

The first CT scan should be completed immediately after admission to identify ICH, which may be less than 6 h or between 6 and 72 h from onset. The secondary CT scan must be done after the onset time at 24 h, mainly to evaluate the haematoma growth and the oedema around the haematoma. The last CT scan can be completed within 10 to 14 days after onset, mainly to evaluate the enlargement or absorption of the haematoma. We define the amount of bleeding as the volume of the haematoma by the chef formula (the volume of haematoma=the maximum diameter of haematoma × wide diameter × CT dimension × CT layer thickness ×*π*/6).

### 5.2. Follow-Up and Outcome Evaluation

We will set up 4 main study visit time points in this study to evaluate the patient's status dynamically. The 4 study visit time points include the following: when the subject is admitted, 24 h after onset, 10 to 14 days after onset, and 90 plus-or-minus 7 days after onset. The researchers must finish the GCS scoring, NIHSS scoring, water drinking test, traditional Chinese medicine pattern assessment form for stroke, etc. The BI index scoring and mRS scoring will be taken at the time point 10 to 14 days after onset and 90 plus-or-minus 7 days after onset. The water drinking test will be administered at all time points except for the first.  Table 3 shows all the detailed information of the follow-up and outcome evaluation.

### 5.3. Primary Outcome

The enlargement percentage of the haematoma measured on the CT scan at the time 24 h after onset is one of the primary outcomes in this study. The definition of haematoma enlargement is a haematoma volume enlarged 33% or increased by 12.5 ml, which is compared with the two CT scans (6-72 h from onset and 24 h after onset or 6-72 h from onset and 10-14 days after onset)

### 5.4. Secondary Outcomes

There are 7 secondary outcomes: (1) GCS scoring scale for consciousness assessment; (2) NIHSS scoring scale for neurological impairment assessment; (3) BI index for living ability assessment; (4) social function activity questionnaire (FAQ) to evaluate the quality of life after stroke; (5) the case fatality rate on 14 days after the onset; (6) the disability rate at the 90 day follow-up; and (7) other general questionnaires to collect information about the blood type and genotype of the patients.

### 5.5. Adverse Event Validation

We record all adverse events during the experiment process in detail, and the adverse events should not be considered as a single symptom. The researchers must assign adverse events into three categories: mild, moderate and severe. Also, the possible causes and the processing method should be written on the case observation chart. If it is uncertain whether the symptom is related to the medicine, it will be recorded in detail. To judge the adverse reactions in clinical trials, we mainly follow these five principles: (1) the rationality of the time sequence of medication and adverse events; (2) adverse events reported of this herbal medicine in the past; (3) the withdrawal effects of this herbal medicine; (4) the results of the remedication after the disappearance of adverse events; and (5) considerations of other confounding factors. Lastly, the researchers will judge and decide the adverse event validation using descriptions of irrelevant, may not be relevant, probably relevant, most likely relevant, and certainly relevant.

### 5.6. Blinding

Before unblinding, only the researchers responsible for the randomized method knew the background grouping data. All the participants in this study, including the principal investigator, researchers, experimental subjects, controllers, and data analyst, will be blinded about the background data. Only in the case of severe adverse events closely related to the treatment will the blind be broken.

### 5.7. Statistical Analyses

The data analysis for this experiment is a comparative analysis of multiple independent sample rates, in accordance with the standard that *α*=0.05 (the size of the first error in hypothesis testing), 1-*β*=0.9 (the efficiency of the expected test), and *δ*=2 (the significant differences, more than 50% of the volume of haematoma and greater than that by 2 ml). In this study, the percentage change in the volume of the haematoma is measured and calculated from the baseline CT scan and the 24 h CT scan. The 7 test centres, researchers, and the CT readers are fitted with a random effect model. Also, the baseline CT scan, time course from onset to CT scan, and interventions during the research are fitted with a fixed effect model. The percentage change rate of haematoma volume will be converted to logarithmic form and normally distributed. The two treatment groups will be analysed comparatively by chi-square distribution with Bonferroni correction.

### 5.8. Data Monitoring and Quality Control

The Data Safety Management Centre (DSM) from the Guangdong Province Hospital of Traditional Chinese Medicine is responsible for the data and safety monitoring. The data inspectors trained by the DSM will complete and submit the physical and chemical examination normal value range table to the DSM. If there is any indication of impropriety, the DSM will stop the trial and the person responsible for the trial must make a detailed description. Annual quality control meetings will be held in the duty hospital, and the researchers from all the participating hospitals must give a report about the trial progress in detail. The patients joining the trial will be registered by key information on the ClinicalTrial.gov online website.

## 6. Discussion

Considering the lack of studies in TCM meeting the standard of RCT, further high-quality RCTs are required [22]. We have proven that the use of herbal medicine would not lead to the enlargement of haematoma within 24 h after onset in a retrospective study in 2015[17], which supports the traditional Chinese therapy of activating blood circulation and removing blood stasis in treating haemorrhagic stroke [15, 16]. However, a prospective, randomized, open, double-blind controlled clinical trial we have conducted previously showed the dangers of using PBS and RBS, which may lead to the enlargement of haematoma within 6 h onset [18]. The past surveys showed that the haematoma became stable and the rate of rebleeding was small [19, 20]. Taking this into account, we speculate that it is safer to use herbal medicines for activating blood circulation and removing stasis after 6 h following onset. Thus, we designed a clinical trial about this herbal medicine as an intervention for acute intracerebral haemorrhage (AICH). We expect to provide a new treatment that will be effective at reducing the fatality and disability of AICH under more rigorous preconditions. The CRRICH-I study has been completed, and we have maintained good partnerships between all cooperative hospitals as well as developing rich experience with this kind of clinical trial. In all, these partnerships guarantee a good foundation for the CRRICH-II study. In this study, we suggested that the probable reason may be a small sample size for effective case analysis, the limitation of the time window, and the difference of the individuals' genotype or serum markers. With the development of precision medical treatment, some specific serum markers and some genotypes have been shown to be risk factors for haematoma enlargement [23]. At present, APOE genotype and von Willebrand factor genotype have been confirmed to be related to the enlargement of ICH haematoma [10, 24, 25]. The question of whether the individual sensitivity to traditional Chinese medicine is related to the serum markers or genotype requires further study. Further defining the relationship between serum markers and genotype will further expand the scope of the CHMF of traditional Chinese medicine and provide evidence supporting its safety.

## Figures and Tables

**Table 1:** The univariate analysis on the haematoma enlargement (%)[17].

Factor	Haematoma enlargement (%)(n=43)	Non-haematoma enlargement (%)(n=213)	Value	p
PBC and RBS herbal				
Not used	19 (44.2)	78 (36.6)		
PBC	2 (4.7)	26 (12.2)	0.181	0.149
RBS	22 (51.2)	109 (51.2)		
PBC and RBS	24 (55.8)	135 (63.4)	0.870	0.390
Leech	22 (51.2)	109 (51.2)	0.00	1.00
Leonurus	24 (58.1)	124 (58.2)	0.085	0.866
Rhizoma	22 (51.2)	109 (51.2)	0.00	1.00

**Table 2:** Groups and Interventions.

**Groups**	**Assigned Interventions**	**Dosage and Taking**
ICH-012	8 herbal medicine (with 2 herbals for activating blood stagnation and expelling blood stasis herbs)	One bag, po., bid for 10 days. Open the medicine bag and take it after mixing with 50-80 ml warm water (Or take by nasal feeding).
ICH-012-II	6-herbal medicine (the same as ICH-012 but without 2 herbals- folium sennae and Snakegourd seed)	
Placebo	Placebo medicine made of starch, bitter-tasting additive, and cyclodextrin	

**Table 3:** The flow table of the follow-up and outcome evaluation of clinical trial.

Objects	Screening form	Visit of 6-72h after onset	Visit of 24h after onset	Visit after onset 10 to 14 days	Visit after onset 90 ±7 days
Demographic data	√				

Medical history	√				

Physical examination	√	√			

CT and CTA	√	√	√	√	

Determination of inclusion or not	√				

Informed consent	√				

Biological specimen	√	√	√	√	

Adjoint pharmacy and treatment	√	√	√	√	

GCS, NIHSS, water drinking test	√	√	√	√	

mRS, BI index and questionnaire			√	√	

Adverse event		√	√	√	√

Summary of the subjects and researches					√

## Data Availability

The data used to support the findings of this study are available from the corresponding author upon request.

## Abbreviations

ICH: Intracerebral haemorrhage
HICH: Hypertensive intracerebral haemorrhage
RCT: Randomized controlled trial
CT: Computed tomography
NIHSS: National Institutes of Health Stroke Scale
GCS: Glasgow Coma Scale
ApoE: Apolipoprotein E
TCM: Traditional Chinese medicine
BI index: Barthel index
FAQ: Function activity questionnaire
DSM: Data safety management
CHMF: Chinese herbal medicine formula
DSTGP: The Department of Science and Technology of Guangdong Province of PRC.
GSTIC: The Guangzhou Science Technology and Innovation Commission of PRC.
GPHTCM: The Guangdong Province Hospital of Traditional Chinese Medicine.
